# The Relationship Between Anxiety Levels, Sleep, and Physical Activity During COVID-19 Lockdown: An Exploratory Study

**DOI:** 10.3389/fpsyg.2021.659599

**Published:** 2021-03-30

**Authors:** Roberta Frontini, Ricardo Rebelo-Gonçalves, Nuno Amaro, Rogério Salvador, Rui Matos, Pedro Morouço, Raul Antunes

**Affiliations:** ^1^Center for Innovative Care and Health Technology (ciTechCare), Polytechnic of Leiria, Leiria, Portugal; ^2^CIEQV - Life Quality Research Centre, Polytechnic of Leiria, Leiria, Portugal; ^3^ESECS, Polytechnic of Leiria, Leiria, Portugal; ^4^Research Unit for Sport and Physical Activity (CIDAF – uid/dtp/04213/2020), University of Coimbra, Coimbra, Portugal; ^5^Sport Science School of Rio Maior (ESDRM), Polytechnic Institute of Santarém, Rio Maior, Portugal

**Keywords:** anxiety-state and trait, coronavirus, coronavirus disease-19 outbreak, coronavirus disease-19, physical activity

## Abstract

Nowadays and worldwide, the attention is focused on coronavirus disease (COVID-19), and its consequences on mental health are yet to be fully understood. It is important to capture differences in anxiety levels among populations, groups, and the gender-related variation. Therefore, the present study had two main purposes: (1) to characterize the levels of state anxiety and trait anxiety by examining gender-related, sleep-related, and physical activity-related variations in a nonrepresentative sample of the Portuguese population during the first weeks of lockdown; and (2) to explore the possible relationship between trait anxiety and state anxiety and the possible role of gender as a moderator. This cross-sectional study comprised 1,332 Portuguese adults (aged 18–55 years old) recruited online during COVID-19 outbreak measures. Participants answered to sociodemographic data and the Portuguese version of the State-Trait Anxiety Inventory (STAI). Gender differences were found in both state anxiety (*p* = < 0.001; *d* = 0.385) and trait anxiety (*p* = < 0.001; *d* = 0.467) with females presenting higher values. People reporting doing more physical activity than usual during COVID-19 lockdown presented lower levels of state anxiety (*p* = < 0.001; *d* = 0.200). People reporting more satisfaction with the quality of sleep presented lower levels of both state anxiety (*p* = < 0.001; *d* = 0.701) and trait anxiety (*p* = < 0.001; *d* = 0.899). Variation associated with the physical activity level (low, moderate, and high) was significantly different among groups in both state anxiety (*p* = < 0.001) and trait anxiety (*p* = < 0.001). When analyzing in more detail separating the levels of physical activity, participants performing moderate and high physical activity showed lower values of state and trait anxiety compared to participants with low physical activity. Participants performing high physical activity also showed lower values of state anxiety compared to participants performing moderate physical activity. Higher levels of trait anxiety were related to higher levels of state anxiety, but this association was not moderated by gender. Interventions aiming to support people psychologically during this outbreak should consider anxiety as well as gender and possible behavioral changes in sleep and physical activity, for example. Health professionals should not only consider the anxiety related to the situation we are living but also address trait anxiety to help overcome COVID-19 psychological consequences.

## Introduction

Global attention is focused on the coronavirus disease (COVID-19) and its physical and psychosocial consequences, particularly on mental health. The World Health Organization (WHO) on the 30th January 2020 declared the new coronavirus disease as a public health emergency of international concern and later as a pandemic (World Health Organization, 2020a). Since then, countries worldwide have implemented a set of exceptional and urgent measures to mitigate the transmission of the virus, particularly through measures of social isolation and quarantine (Martins, 2020; World Health Organization, 2020b). The Portuguese government declared a state of emergency on March 18, and, consequently, a set of preventive public health measures were gradually implemented (Ribeiro et al., 2020).

Although these measures are vital to prevent the spread of the virus and may have a positive effect in protecting peoples’ physical health, it may also have consequences on mental health, for instance, higher levels of anxiety (Cao et al., 2020; Huang and Zhao, 2020), depression, or even stress (Roma et al., 2020; Hawes et al., 2021). In fact, these preventive measures might induce the separation of significant relatives, the perception of isolation, the loss of freedom, and the demanding restructure of a new lifestyle (Jeong et al., 2016; Brooks et al., 2020). The fact that we are living in an unprecedented situation entails several feelings of fear and uncertainty that, along with the aforementioned consequences of the application of preventive measures, may enhance anxiety levels.

Taking into consideration these possible negative consequences, the WHO emitted a set of deliberations in order to minimize the increasing levels of stress and anxiety that may arise in these times of uncertainty (World Health Organization, 2020e). These measures comprise the need to maintain family routines and to seek a healthy lifestyle, namely, through the practice of regular physical activity (PA) as well as the adoption of healthy eating and healthy sleep habits (World Health Organization, 2020c). This is particularly important considering that studies conducted during the first COVID-19 lockdown found that sleep habits changed during quarantine, with people having poor quality of sleep (Chouchou et al., 2020). Changes in sleep quality may be due to anxiety and feelings of uncertainty typical of this period (Ingram et al., 2020; Morin and Carrier, 2020).

It has been suggested that changes in daily routines during lockdown may lead to an increase in sedentary behaviors and anxiety levels (Chen et al., 2020), highlighting the importance of physical activity in coping with such consequences.

Regular physical activity is proven to reduce symptoms of anxiety and improve mental health and well-being (Stubbs et al., 2017; WHO 2021 guidelines). A study of Chouchou et al. (2020) has also found that lack of physical activity and poor-quality sleep during COVID-19 lockdown are related to a decrease in well-being. Moreover, it is important to note that mental health is vital for preventing illnesses (Hammami et al., 2020; Woods et al., 2020). Considering the current pandemic, increasing the immune system and fighting for better mental health are of utmost importance (Woods et al., 2020).

Although the impact of physical and social isolation has been previously studied (Gammon and Hunt, 2018; Sharma et al., 2020), with several papers having been published regarding the consequences of COVID-19 on physical and mental health in different countries (Antunes et al., 2020; Cao et al., 2020; Ho et al., 2020; Morin and Carrier, 2020), its extents are still very recent and much research is necessary. For instance, it is vital to map a range of antecedent factors (e.g., gender) and understand its role. Regarding physical activity, several studies consistently showed that men usually present higher levels of physical activity when compared to women (Azevedo et al., 2007; Hallal et al., 2012; Chatfield, 2015; Guthold et al., 2018). As regards anxiety levels, past research before COVID-19 pandemic consistently found that women present higher levels of anxiety compared to men (McLean and Anderson, 2009; McLean et al., 2011), but it is necessary to understand if these relationships are still observed during COVID-19 pandemic or if the situation we are currently living in affects both genders in the same way. Understanding gender-associated differences may be important considering that policy responses had not yet addressed the gendered impacts of disease outbreaks (Smith, 2019; Galasso et al., 2020). For instance, past literature has consistently found a relationship between anxiety trait and anxiety state (Horikawa and Yagi, 2012). However, we do not know if this relationship persists in an unprecedented moment such as the one lived during COVID-19. Moreover, and considering the role played by gender (McLean et al., 2011), it may be interesting to understand if the relationship between the two types of anxiety may be moderated by gender.

The aim of this two-folded study was (1) to characterize the levels of anxiety (trait and state) considering gender, quality of sleep, and physical activity in a sample of Portuguese adults, during the period of COVID-19 lockdown, and (2) to explore the moderator role of gender in the association between trait anxiety and state anxiety.

## Materials and Methods

### Study Design and Procedures

This was a cross-sectional study conducted in the period between April 1st and 15th. During this period, a state of emergency was decreed by the Portuguese president. This study involved adults and comprehended a set of self-reported questionnaires assessing different domains of an individual’s behavior and feelings toward the lockdown period of COVID-19.

Google forms were used as a survey platform for electronic distribution. The assumptions of nonduplication of response were taken into account, namely, by limiting to one response per account. Respondents were required to sign into Google. Social media and newspapers were used to advertise and recruit possible volunteers who received no compensation for their participation. Each questionnaire assessed four domains: sociodemographic data, physical activity levels, state anxiety, and trait anxiety levels.

The survey with sociodemographic questions was previously developed and reviewed by four experts in the area of exercise and psychology. The other domain assessment comprised validated instruments for the Portuguese population. Procedures followed standards for research in sports medicine and were performed according to the Declaration of Helsinki.

### Participants

Subjects were only eligible if they were aged over 18 years old. The sample was composed of a total of 1,332 respondents (35.02 ± 10.19 years of age), ranging from 18 to 55 years of age, from which 932 (69.99%) were women and 399 (30%) were men, and only one respondent preferred not to specify. Respondents were fully informed regarding the nature of the study, the procedures on data recording, and the voluntary nature of their participation. They were also informed that they could withdraw from the study at any time. Subjects provided their consent before the survey’s completion, and anonymity was guaranteed. Respondents took an average time of 14 min to complete the survey.

### Variables

#### Sociodemographic Characterization

In the sociodemographic survey, respondents were invited to answer simple questions regarding age, gender, marital status, living status during confinement, and academic level. Then, they were asked to self-report about sleep duration (inferred from regular bedtime and getting up) and quality of the sleep, the amount and frequency of food intake, and the time spent watching pandemic-related news.

#### Anxiety

The Portuguese version (Silva, 2003) of the State-Trait Anxiety Inventory (STAI-state, STAI-trait) (Spielberger et al., 1983) was used. This questionnaire is composed of two blocks (Form 1 and Form 2) of 20 statements, evaluated in a four-point Likert scale. Form 1—STAI-State evaluates transient or temporary anxiety, i.e., the anxiety that the person is feeling at the present moment. Form 2—STAI-Trait assesses dispositional or general anxiety. The score is generated by the sum of the 20 items for each scale. Higher levels correspond to higher anxiety levels. Examples of items include “I’m worried” (state) and “I often feel that I’m not able” (trait). Internal consistency in this study proved to be good (state *α* = 0.93; trait *α* = 0.93).

#### Physical Activity

The Portuguese validated version of The International Physical Activity Questionnaire (IPAQ-short form) was used to assess PA (Craig et al., 2003). This questionnaire is composed of four questions related to specific types of PA, e.g., walking and moderate and higher activities, in terms of the frequency and duration of each specific type of activity, and the time spent seated per day in a week. The obtained data by the IPAQ are then converted into MET-min/week (metabolic equivalent) through the calculation of the marked minutes per week in each category of activities by their specific metabolic equivalent.

#### Data Analysis

The Statistical Package for the Social Sciences (IBM SPSS, version 26.0; IBM SPSS, Armonk, NY, United States) was used to perform the data analysis. Descriptive statistics were computed for all sociodemographic and study variables. Counts (and proportions), means, standard deviations (sd), 95% confidence interval (95% CI), and medians (interquartile range, IQR) were computed to describe both categorical and continuous variables for the total sample. Comparison analyses were performed to assess the differences between gender (male vs. female), the differences between people who performed more PA compared to people who did not perform more PA during COVID-19 lockdown, and the differences between people who were not satisfied with the quality of the sleep compared to people who were satisfied with the quality of the sleep. Independent samples t-tests for continuous variables were performed. Analysis of variance (ANOVA) was used for between-group (PA levels—low, medium, and vigorous) comparisons. Cohen’s d analyses were performed to evaluate the effect size. A mediation model was performed to assert if the relationship between trait anxiety and state anxiety was moderated by gender (Model 1; Hayes, 2013). The moderation was estimated using PROCESS (Hayes, 2013), an SPSS macro for path analysis-based moderation and mediation analysis. Anxiety trait was used as the independent variable and anxiety state as the dependent variable. Gender was the moderator. A bootstrapping procedure was used (with 10,000 resamples). Significance was set at the 0.05 level.

## Results

The sample characteristics are presented in Table 1. Most of the sample was married and living in social isolation at home, not working, with other people.

**Table 1:** Summary of descriptive statistics (*n* = 1,332).

	*n* (%)	Mean	
		Mean ± SD	(CI 95%)
Age (years)		35.02±10.19	(34.47 to 35.57)
**Gender**			
Men	399 (30.0)		
Women	932 (69.9)		
Preferred not to specify	1 (0.1)		
**Marital Status**			
Single	614 (46.1)		
Married	591 (44.4)		
Separated	16 (1.2)		
Divorced	98 (7.4)		
Widower	4 (0.3)		
Other	9 (0.7)		
**Living status—COVID 19**			
In social isolation at home, not working and alone	36 (2.7)		
In social isolation at home, not working, with other people	443 (33.3)		
Working out in full-time	134 (10.1)		
Working out in part-time	100 (7.5)		
Teleworking at home, alone	65 (4.9)		
Teleworking at home, with other people	570 (41.3)		
Home quarantine	3 (0.2)		
**Academic level**			
Elementary	96 (7.2)		
Secondary	245 (18.4)		
Professional	103 (7.7)		
Superior	888 (66.7)		
**Sleep quality satisfaction**			
Yes	777 (58.3)		
No	555 (41.7)		
**Higher food frequency**			
Yes	612 (45.9)		
No	720 (54.1)		
**Time spent watching, reading, or listening to the news about Coronavirus**			
None	10 (0.8)		
Less than 1 h	497 (37.3)		
Between 1 and 3 h	709 (53.2)		
More than 3 h	113 (8.5)		
Another option	3 (0.2)		

Gender-associated variation showed significant differences among groups in both state anxiety and trait anxiety (Table 2) with females presenting higher values compared to males. Variations associated with PA were also found in state anxiety with people reporting doing more PA than usual during COVID-19 lockdown presenting lower levels of state anxiety. Variation associated with satisfaction with sleep quality was also found. In fact, in both state anxiety and trait anxiety, people reporting more satisfaction with the quality of their sleep presented lower levels of both state anxiety and trait anxiety. According to Cohen’s interpretation (Cohen, 1988), small to large effects of gender and quality of sleep were present in both trait and state anxiety.

**Table 2:** Variation in state anxiety and trait anxiety associated with gender, PA, and quality of sleep.

		Anxiety (trait)	*P*	Effect size	Anxiety (state)	*P*	Effect size
Gender	Male (*n* = 399)	35.27 ± 9.88	<0.001	0.385	41.51 ± 10.54	<0.001	0.467
	Female (*n* = 932)	39.19 ± 10.32			46.62 ± 11.10		
Do you do more PA than usual?	Yes (*n* = 385)	37.34 ± 9.77	0.128	0.092	43.51 ± 10.81	0.001	0.200
	No (*n* = 947)	38.29 ± 10.55			45.74 ± 11.26		
Are you satisfied with the quality of your sleep?	Yes (*n* = 777)	35.16 ± 9.08	<0.001	0.701	41.27 ± 9.74	<0.001	0.899
	No (*n* = 555)	42.01 ± 10.67			50.45 ± 10.85		

Variation associated with the PA level showed significant differences among groups in both state anxiety and trait anxiety (see Table 3). Participants who were classified as performing moderate (Group 2) or high (Group 3) PA showed lower values of both state anxiety and trait anxiety compared to participants who were classified as performing low PA (Group 1). Participants who were classified as performing high PA (Group 3) also showed lower values of state anxiety compared to participants who were classified as performing moderate PA (Group 2).

**Table 3:** Comparison between the IPAQ categories, anxiety levels (*n* = 1,332).

	IPAQ Category 1 Low (*n* = 25) Group 1	IPAQ Category 2 Moderate (*n* = 665) Group 2	IPAQ Category 3 High (*n* = 242) Group 3		
	Mean ± SD	Mean ± SD	Mean ± SD	*F*	*Post-hoc*
Anxiety (state)	46.99 ± 11.59	44.79 ± 10.99	42.59 ± 10.38	12.69^**^	1 > 2 1 > 3 2 > 3
Anxiety (trait)	39.87 ± 10.79	37.51 ± 9.91	36.15 ± 10.22	11.75^**^	1 > 2 1 > 3

** *p* < 0.001.

### Moderation Analyses

**Figure 1:**
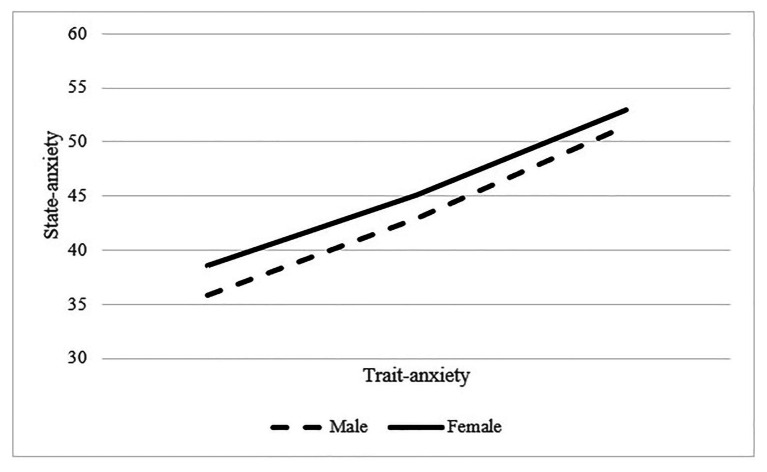
The associations between trait-anxiety and state-anxiety.

To test the moderating effect of gender on the associations between trait anxiety and state anxiety, path analysis-based moderation was performed. Next, using ModGraph (Jose, 2013), this significant interaction was plotted (see Figure 1). Results showed that higher levels of trait anxiety were related to higher levels of state anxiety, but this association occurred in both males and females.

## Discussion

The aim of this study was to characterize the levels of anxiety (trait and state) considering gender, quality of sleep, and physical activity in a subsample of Portuguese adults during the period of COVID-19 lockdown, and to explore the moderator role of gender in the association between trait anxiety and state anxiety. Females presented higher values of both state and trait anxiety. People reporting doing more PA than usual during COVID-19 lockdown presented lower levels of state anxiety. People reporting more satisfaction with the quality of their sleep presented lower levels of both state anxiety and trait anxiety. Variation associated with the PA level was significantly different among groups in both state anxiety and trait anxiety. Participants performing moderate and high PA showed lower values of state and trait anxiety compared to participants with low PA. Participants performing high PA also showed lower values of state anxiety compared to participants performing moderate physical activity. Higher levels of trait anxiety were related to higher levels of state anxiety, but this association was not moderated by gender.

Higher anxiety levels, both trait and state, were found for female participants in our study. These results are in line with previous evidence in the literature (McLean and Anderson, 2009; McLean et al., 2011); females tend to present higher levels of anxiety. This has also been verified in recent studies carried out in the current context of lockdown caused by the COVID-19 pandemic (Antunes et al., 2020), even in other countries such as China (Hou et al., 2020; Pieh et al., 2020) or Austria (Xiang et al., 2020). These results seem to indicate that females have a greater predisposition to report higher levels of anxiety, regardless of the global context, stressing the importance of adopting specific measures in between genders. Typically, in health centers, hospitals, and other contexts, professionals have more warns of gender difference. Considering the situation of lockdown and isolation that we are again experiencing, families must be aware of this possible difference and the possibility of an increase in anxiety levels at this stage, an increase that may be more pronounced in the female gender.

When the comparison was performed according to the satisfaction with the quality of sleep, we found that individuals who reported being satisfied with the quality of their sleep, during the lockdown period, had lower levels of anxiety (both trait and state). Thus, having an appropriate sleep hygiene may be necessary for helping to lower the anxiety levels experienced during the lockdown, as sleep routines may become less rigid, more flexible, or even chaotic. Considering the protective role that sleep seems to have for anxiety levels and stress (Wunsch et al., 2017), our results emphasize the importance of having a good quality of sleep.

Our results also showed that participants reporting an increase in PA levels during lockdown presented lower levels of state anxiety. In more detail, by examining the results according to the amount of PA performed, based on the three categories recommended by the IPAQ questionnaire, we found that people who reported practicing a greater amount of PA during the lockdown (Group 3—high PA) were those who reported lower levels of anxiety (state and trait). These indicators seem to underline the evidence in the literature regarding the potential role of PA (Antunes et al., 2020; Hammami et al., 2020; Ingram et al., 2020), as well as the importance of sleep quality (Antunes and Frontini, 2020; Ingram et al., 2020), in preventing the psychosocial impact of lockdown. Thus, our results advocate that people who are most concerned with following health-promoting behaviors, for example, by practicing more PA and assuming healthier sleep habits, may be simultaneously those who have lower levels of anxiety, state anxiety in particular. This is in line with the recommendations of the health authorities to prevent mental health problems in this period (World Health Organization, 2020d; Portuguese Psychologists Order, 2021). Specifically, regarding PA, these results reinforce, once again, the key role of PA practice, specifically in this context (Di Sebastiano et al., 2020; Maugeri et al., 2020), underlining the importance of creating strategies for its promotion and implementation in a more generalized way in the population and according to the recommendations for specific situations like this pandemic (Hammami et al., 2020).

In fact, and in Portugal specifically, there have been various initiatives promoted by individuals and institutions (private and public) using the media and social networks. Future studies would benefit from understanding the impact of private and public initiatives using media and social networks on promoting appropriate behaviors to cope with social distancing and lockdowns. Furthermore, it is important to highlight what has already been stated and empirically found in literature: that PA should be widespread and promoted. However, it should be monitored by adequate professionals. Moreover, considering that the monitorization of PA may not be possible in this period, home-based PA programs must also take these differences into account, also considering the different motor skills level between gender in order to prevent feelings of frustration and, thus, anxiety especially in women.

Finally, in the examination of the moderating role of gender in the association between state anxiety and trait anxiety, we found that higher levels of trait anxiety are related to higher levels of state anxiety, which is in line with past literature and the theoretical model of anxiety (Matthews and Deary, 1998). As stated in the literature, individuals with higher levels of trait anxiety will be likely to perceive more situations as threatening and, thus, will experience more intense anxiety states compared to individuals with low trait anxiety (Spielberger, 1972). However, in our study, this association occurs equally in both genders. This result seems to indicate that also in specific situations like this pandemic, where there is a tendency for higher levels of anxiety (Paredes et al., 2021; Paulino et al., 2021), the relationship remains and is independent of gender. Therefore, those who have higher trait anxiety are then those who appear to be more vulnerable to increased state anxiety, whether they are men or women. This result should lead health authorities to pay attention to this aspect, namely, paying special consideration to people who have a higher level of anxiety because they seem to be more vulnerable also in this period.

The results of this study are in line with other research studies carried out during COVID-19 pandemic (Di Sebastiano et al., 2020; Sang et al., 2021) that have given important indications crucial for the health authorities in case of the need for new confinements of the population, which recently happened all over the world. We, therefore, consider that the application of measures to restrict or limit contacts as a preventive factor of contagion (from COVID-19 or other diseases) should be accompanied by an increase in health and PA literacy so that people develop adaptive strategies to deal with the “normal” consequences of this physical distance. Thus, our study underlines the role of regular PA practice, as well as the maintenance of sleep routines that allow the preservation of its quality. On the other hand, this study seems to suggest the importance of strategies to promote mental health and minimize the psychosocial impact of confinement. These strategies must be specific and individualized. Moreover, identifying earlier those who may have higher levels of anxiety during a lockdown situation and/or a state of social isolation is of utmost importance. The results of our study suggest that women, people not practicing the recommended PA, and people with higher anxiety traits may be those in higher need.

The current study presents the following limitations that should be noted: (1) the cross-sectional design of the study (a longitudinal design is needed to provide solid and causal evidence for the direction of these associations); (2) no validated questionnaire was used to assess sleep quality; and (3) the sample was one of convenience and recruited on the Internet and which does not allow the generalization of results. Future studies should resort to more objective measures (and not just self-report) to monitor the levels of PA practiced or the quality of sleep.

## Conclusion

A period of social lockdown is still necessary for the protection of community health. We live in times of uncertainty, but the scientific community consistently found the importance of physical isolation to reduce the risk of contamination. However, physical isolation may have long-lasting negative consequences, especially for mental health. The definition and effectiveness of intervention strategies for promoting the quality of life and well-being of people during these periods of physical isolation could be enhanced by considering the role of psychological dimensions and lifestyle habits as well as the relationship with PA. The results of the present study may contribute to creating strategies for an improvement in mental health during COVID-19 by enhancing the importance of PA and good sleep quality. Getting physically active during this pandemic is of utmost importance and has been demonstrated in several studies. However, there is a lack of strategies and interventions to promote it effectively.

To sum up, our results highlight the importance of identifying which groups may face more difficulties during COVID-19 pandemic, future waves, and other pandemics that may come in the future. Identifying these vulnerable groups will help in targeting better intervention strategies.

## Data Availability Statement

The raw data supporting the conclusions of this article will be made available by the authors, without undue reservation.

## Ethics Statement

Ethical review and approval was not required for the study on human participants in accordance with the local legislation and institutional requirements. The patients/participants provided their written informed consent to participate in this study.

## Author Contributions

RA was the leader of the research group that conducted the study. RF, RA, RR-G, NA, RS, RM, and PM contributed to the conception and design of the study. RF, RA, and RR-G organized the database. RF and RA performed the statistical analysis and wrote the first draft of the manuscript. NA, RS, RM, PM, and RR-G reviewed and edited the first draft. All authors contributed to the article and approved the submitted version.

### Conflict of Interest

The authors declare that the research was conducted in the absence of any commercial or financial relationships that could be construed as a potential conflict of interest.
